# The interpretation of disease phenotypes to identify TSE strains following murine bioassay: characterisation of classical scrapie

**DOI:** 10.1186/1297-9716-43-77

**Published:** 2012-11-01

**Authors:** Katy E Beck, Christopher M Vickery, Richard Lockey, Thomas Holder, Leigh Thorne, Linda A Terry, Margaret Denyer, Paul Webb, Marion M Simmons, John Spiropoulos

**Affiliations:** 1 Animal Health and Veterinary Laboratories Agency, Addlestone, Surrey KT15 3NB, United Kingdom; 2 University of Southampton, University Road, Southampton, SO17 1BJ, United Kingdom; 3 Université de Genève, 1 Rue Michel Servet, 1211, Geneva, 4, Switzerland

## Abstract

Mouse bioassay can be readily employed for strain typing of naturally occurring transmissible spongiform encephalopathy cases. Classical scrapie strains have been characterised historically based on the established methodology of assessing incubation period of disease and the distribution of disease-specific vacuolation across the brain following strain stabilisation in a given mouse line. More recent research has shown that additional methods could be used to characterise strains and thereby expand the definition of strain “phenotype”. Here we present the phenotypic characteristics of classical scrapie strains isolated from 24 UK ovine field cases through the wild-type mouse bioassay. PrP^Sc^ immunohistochemistry (IHC), paraffin embedded tissue blots (PET-blot) and Western blotting approaches were used to determine the neuroanatomical distribution and molecular profile of PrP^Sc^ associated with each strain, in conjunction with traditional methodologies. Results revealed three strains isolated through each mouse line, including a previously unidentified strain. Moreover IHC and PET-blot methodologies were effective in characterising the strain-associated types and neuroanatomical locations of PrP^Sc^. The use of Western blotting as a parameter to define classical scrapie strains was limited. These data provide a comprehensive description of classical scrapie strain phenotypes on isolation through the mouse bioassay that can provide a reference for further scrapie strain identification.

## Introduction

Classical scrapie, which affects sheep and goats, is the prototypic transmissible spongiform encephalopathy (TSE), a family of fatal neurodegenerative diseases that also affect humans and other mammals. Histopathologically, classical scrapie manifests primarily as spongiform change and gliosis in the central nervous system (CNS) of affected animals [1]. The principal, and possibly only, causative agent in TSEs is the proteinaceous infectious particle, termed prion [1,2], created by the conversion of host encoded prion protein (PrP^C^) to a pathogenic isoform termed PrP^Sc^[3]. PrP^Sc^ is detectable by immunochemical methods, can precede clinical and histopathological signs and is widespread in the CNS of classical scrapie infected animals at end stage disease [4].

A fundamental trait of prions is their ability to manifest as distinct strains that demonstrate characteristic and reproducible phenotypes when passaged in a given species [5]. Classical scrapie is considered to comprise of numerous strains. For several decades panels of inbred mice have been utilised as valuable research tools to investigate the biological properties of TSE isolates and to distinguish strains, which give rise to characteristic incubation periods (IP) and patterns of disease specific vacuolation across the brain when inoculated into mice via a consistent route [5]. The wild-type mouse lines typically employed for bioassay are RIII, C57BL/6 and VM. RIII and C57BL/6 lines share the same *Prnp* amino acid sequence (*Prnp*^*a*^) whilst VM mice (*Prnp*^*b*^) differ at codons 108 and 189 [6]. This difference in genotype affects the phenotype of a transmitted source [7]. Indeed from a single source, two distinct mouse-passaged strains are usually isolated in the two murine genotypes.

Traditional strain typing methodology dictates that an isolate is independently passaged through the C57BL/6 and VM line until the strain is stabilised, as measured by a non significant difference in the IP between consecutive serial passages [8] and then passaged into panels of C57BL/6, VM and C57BL/6 × VM mice for final strain characterisation. This is a lengthy experimental process, particularly because the first inter-species passage can be associated with prolonged IP and lower attack rates compared with subsequent intra-species passages [9,10], a property called the species or transmission barrier.

The identification of PrP^Sc^ as the probable disease-causing agent has augmented the development and application of alternative methods for the diagnosis and characterisation of TSE strains. These methods can also be applied to murine models and as such represent additional parameters to characterise strain phenotype. They include immunochemical methods that detect PrP^Sc^ in tissue sections, i.e. immunohistochemistry (IHC) [10-12], paraffin-embedded tissue (PET)-blot [13,14] and histoblot [15], those which characterise its molecular profile, including electrophoretic mobility of PrP^Sc^ following proteinase K digestion and ratio of PrP^Sc^ glycoforms [16] and assays that measure differences in PrP^Sc^ sensitivity to proteinase K, and its conformational properties and stability (conformation-dependent immunoassay) [17].

Ultimately the use of alternative methodologies in conjunction with the traditional bioassay approach can expand the definition of strain phenotype and crucially, if defined phenotypic parameters are identifiable at earlier passages, would reduce the time and number of mice required to perform strain characterisation. In accordance with this hypothesis we have previously reported that PrP^Sc^ deposition patterns indicative of specific strains were identified following first passage of ovine classical scrapie isolates to wild-type mice [10,18,19].

The purpose of this study was to devise a methodological approach by which a large number of classical scrapie sources could be characterised following mouse bioassay. The phenotypic properties arising from 24 classical scrapie field sources that have undergone full strain typing through either the C57BL/6, VM, or both mouse lines are reported, according to PrP^Sc^ deposition patterns revealed by IHC and PET-blot analysis and by Western blot profiling in addition to standard strain typing methodology. Using the approach suggested here we identify and characterise several scrapie strains and suggest how the strain of TSE sources can be determined without the requirement for a full standard bioassay protocol.

## Materials and methods

### Generation of primary isolates

Primary isolates were generated within two projects where wild-type mouse bioassays were initiated for cases of natural, ovine classical scrapie representing several breeds and PrP genotypes (Table 1). These two initial studies (study 1 and study 2) differed in their sampling period: 1996–1999 or 1998–2002, and in the ovine tissue used to produce the brain homogenate for primary isolation: 10% (w/v) obex homogenate or a 10% (w/v) homogenate prepared from equal parts of obex, cerebellum and frontal cortex, respectively. Primary isolates were generated by inoculating brain homogenate (10% (w/v) ovine brain in normal saline) derived from each individual clinical case into 20 C57BL/6, 20 RIII and 20 VM mice, 20 μL via intra-cerebral and 100 μL via intra-peritoneal routes. Mice were euthanized using carbon dioxide when a pre-determined clinical endpoint had been reached or due to other welfare considerations as described previously [19]. Brains were cut parasagittally to give two unequal portions, the larger of which (approx 2/3 of the brain) was fixed in 10% neutral buffered formalin for histological assessment. The remaining brain material was frozen at −80°C for further transmission experiments or biochemical analysis. All work was carried out in accordance with the Animals (Scientific Procedures) Act 1986 under Home Office project license 70/6310.

**Table 1:** Details of field ovine classical scrapie sources used for bioassay

**Inoculum code**	**Genotype**	**Sheep breed**	**Year collected**
2*	VV:RR:QQ	Clun X^§^	1998
5	AV:RR:QQ	Swaledale	1996
8	VV:RR:QQ	Welsh Mountain X	1997
19	AA:RR:QQ	Mule	1997
19*	AA:RR:QQ	Welsh Mountain X	1998
20	AV:RR:QQ	Mule	1997
32*	AV:RR:QQ	Welsh X Cheviot	1998
41*	VV:RR:QQ	Bleu du Maine	1998
42*	VV:RR:QQ	Bleu du Maine	1999
53	AA:RR:QQ	Suffolk	1997
55	AA:RR:QQ	Suffolk X Mule	1997
59	AA:RR:QQ	Suffolk X	1997
72	AA:RR:QQ	Suffolk	1998
73	VV:RR:QQ	Crossbred	1998
77	VV:RR:QQ	Polled Dorset	1998
80	AA:RR:QQ	Mule	1999
81	VV:RR:QQ	Charollais X	1999
81*	AV:RR:QQ	Bleu du Maine	2000
84*	AV:RR:QQ	Easy care	2000
85*	AV:RR:QQ	Easy care	2000
93*	AV:RR:QQ	Swaledale	2000
103*	VV:RR:QQ	Welsh X Cheviot	1998
104*	VV:RR:QQ	Welsh X Cheviot	1998
111*	AV:RR:QQ	Swaledale	2000

*indicates that the sample originated in study 2.

^§^X indicates a cross bred animal where the main phenotypic characteristics were contributed by the breed indicated. The flocks included in this study were commercial holdings and the breeding status was not always well established.

### Preparation of inocula for serial passage

Mice were monitored and euthanised, and tissue collected and stored as described above for generation of primary isolates. During serial passage of each scrapie source, the portion of frozen brain of the first C57BL/6 and VM mouse whose fixed tissue was identified histopathologically as TSE positive was homogenised with normal saline (1% w/v) and inoculated via the intra-cerebral route into a panel of 10 mice of the same line (20 μL per mouse). Twenty four classical scrapie sources underwent full bioassay. Twenty nine transmissions are reported here because five sources were characterised through both the C57BL/6 and VM mouse lines. It was anticipated that two serial passages (denoted 2^nd^ and 3^rd^ passage) would be adequate to reach stabilisation following which the 4^th^ “characterisation” passage into 10 C57BL/6, 10 VM and 10 C57BL/6 × VM mice would ensue. Where IP did not stabilise and/or if the transmission rate did not reach 100% for each panel of mice, additional serial passages were carried out before final characterisation of the agent. All analyses were carried out on mice at final passage which was the passage used for strain characterisation.

### Histopathological and immunohistochemical analysis

Fixed brain tissue was cut at four coronal levels to reveal caudal medulla, rostral medulla, midbrain, thalamic and frontal levels, required for lesion profiling as detailed previously [19]. TSE diagnosis was made based on the presence of characteristic neuropil vacuolation on haematoxylin and eosin (H&E) stained sections, the severity of which was semi-quantified on a scale of 0–5 or 0–3, for 9 grey matter areas and 3 white matter areas respectively, and then plotted to produce lesion profiles (LP) [20]. Profiles were constructed from at least five clinically and histopathologically positive mice to be considered reliable [21].

For characterisation of the type and neuroanatomical location of PrP^Sc^ associated with each strain identified, IHC was performed with the rabbit polyclonal antibody Rb486 using published methodology [22]. IHC slides were grouped together by inoculum and according to their IP and LP properties. Each group underwent blind analysis. The major PrP^Sc^ deposition types detected were defined as granular, aggregates, plaques, intraglial, punctate, intraneuronal, perineuronal and linear. The predominant deposition types identified across the slides within a given group were recorded onto brain maps of medulla, midbrain, thalamic and frontal coronal sections of the brain as described previously [10]. All available neuroanatomical areas were considered for IHC mapping as opposed to the specific areas in which disease specific vacuolation is scored.

### Western blotting

Tissues were extracted and analysed by Bio-Rad TeSeE™ Western blot (Bio-Rad Laboratories Ltd, Hemel Hempstead, UK). Briefly, 20% (w/v) tissue homogenates were treated with proteinase K (PK) before alcohol precipitation. After centrifugation pellets were solubilised in Laemmli buffer and proteins separated on 12% Bis/Tris gels, electrotransferred to membrane, blocked with 5% (w/v) bovine serum albumin and labelled with biotinylated Sha31 anti-prion antibody [23]. Following addition of streptavidin peroxidase, immunoreactivity was visualised using ECL Western blotting detection reagents (GE healthcare, Chalfont St Giles, UK). PrP^Sc^ bands were quantified using Quantity One software (Bio-Rad Laboratories Ltd) and the relative band intensity (%) of each PrP^Sc^ glycosylation state calculated.

### PET-blot

Paraffin-embedded tissue (PET) blots were performed as described previously [24] with minor modifications: tissues were sectioned at 3 μm and an additional re-hydration step of 25% propan-2-ol following 50% propan-2-ol was performed. Larger membranes were used, therefore the entire process was carried out in individual vessels (crystallising dishes with Petri dishes as lids). Mouse monoclonal anti-PrP antibody 2 G11 (AbD Serotec, Oxford, UK) was used at a concentration of 2 μg/mL. Samples were treated with PK (60 μg/mL for 16 h at 55°C) prior to application of primary antibody. All other conditions were as described.

## Results

### Conventional bioassay parameters

Of 87 transmissions (29 transmissions to C57BL/6, VM and C57BL/6 × VM mouse lines) attack rates for 74 transmissions were 90-100%. For 10 transmissions attack rates were 70% or greater. These are high attack rates as expected at this stage of passaging with very few intercurrent deaths. The remaining three transmissions had lower attack rates as follows: inoculum 53; 40% in C57BL/6 × VM mice (following stabilisation through the VM line), inoculum 59; no transmissions in C57BL/6 or C57BL/6 × VM mice (following stabilisation through the VM line).

#### C57BL/6 mouse line

**Figure 1:**
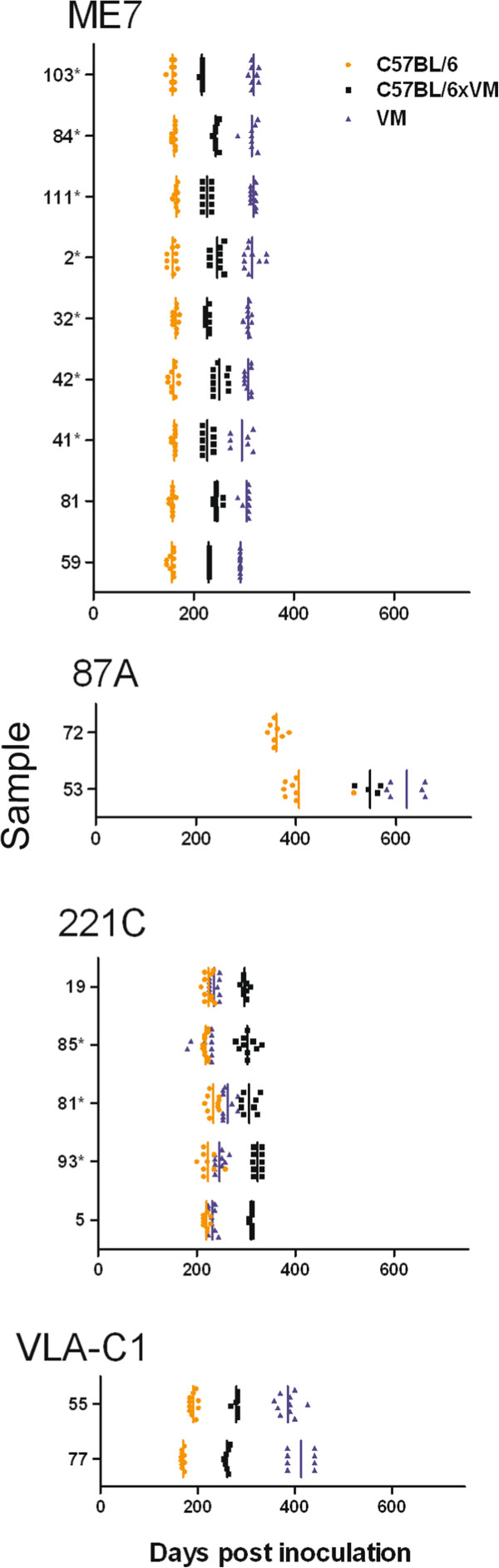
**Incubation periods (days post inoculation) of C57BL/6, VM and C57BL/6 × VM mice, following stabilisation of the agent through the C57BL/6 line. **Samples with comparable IP have been grouped together. For each mouse line the IP for each individual mouse is represented along with the inoculum mean (vertical bar). (*) indicates that the sample originated in study 2.

Figure 1 shows the IP of 18 classical scrapie transmissions following stabilisation through the C57BL/6 line and final characterisation in C57BL/6, VM and C57BL/6 × VM mouse lines. Only mice that were clinically and histopathologically positive were included in the analysis. Sources were grouped according to the strains which they represented according to this phenotypic parameter [7,9,25]. Results show that both the absolute IP as well as the relative IP between mouse lines for a given source differed according to scrapie strain. The principle strains observed were ME7, 87A and 221C. With respect to IP, a group consisting of two sources (of different ovine PrP genotype), designated VLA-C1, did not resemble any previously identified strains from natural sources of classical scrapie.

**Figure 2:**
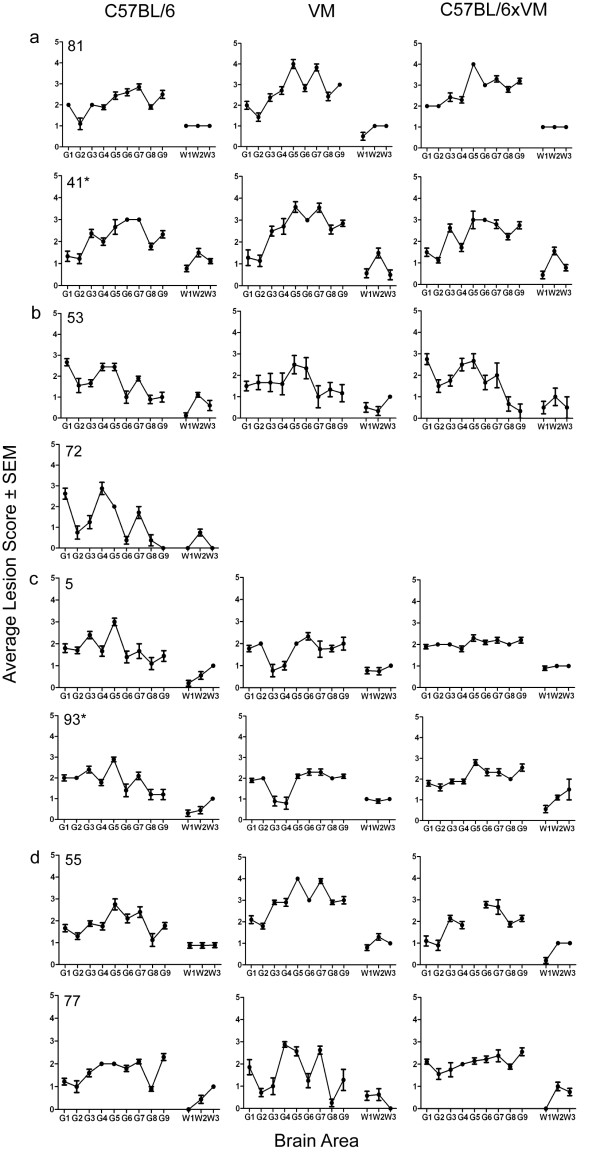
**Lesion profiles of C57BL/6, VM and C57BL/6 × VM mice, following stabilisation of the agent through the C57BL/6 line. **In (**a**) examples of isolates displaying the ME7 LP are shown. In (**b**), (**c**) and (**d**) examples of isolates displaying the 87A, 221C and VLA-C1 LP, respectively, are shown. (*) indicates that the sample originated in study 2. Only clinically and histopathologically positive animals were included in the profile. Error bars indicate standard error of the mean.

Corresponding LP are shown in Figure 2. For each strain identified according to IP, two LP, each from independent sources are presented to demonstrate the degree of heterogeneity that can be observed between LP of the same strain and where possible, represent a source which originated under study 1 and one which originated under study 2. All profiles represent fourth passage data with the exception of isolate 77 which had not fully stabilised at third passage and so was serially passaged again before final characterisation. For source 72 only the C57BL/6 line gave at least 5 clinically and histopathologically positive mice.

Of the two sources that were grouped as VLA-C1 based on IP data, the LP from source 55 following characterisation in all three mouse lines closely resembled the ME7 LP (compare Figure 2d with Figure 2a). However the IP in all mouse lines were prolonged with respect to the predicted IP of ME7. Following serial passage of source 77 in C57BL/6 mice the IP stabilised to a value that is indicative of ME7 (Figure 1). However, the IP from the C57BL/6 × VM and particularly from the VM mice were unusually prolonged. In addition LP from the C57BL/6 mice was indicative of ME7 whilst LP from the C57BL/6 × VM mice resembled those of 221C (Figure 2). In contrast VM generated LP did not resemble any previously identified LP.

#### VM mouse line

**Figure 3:**
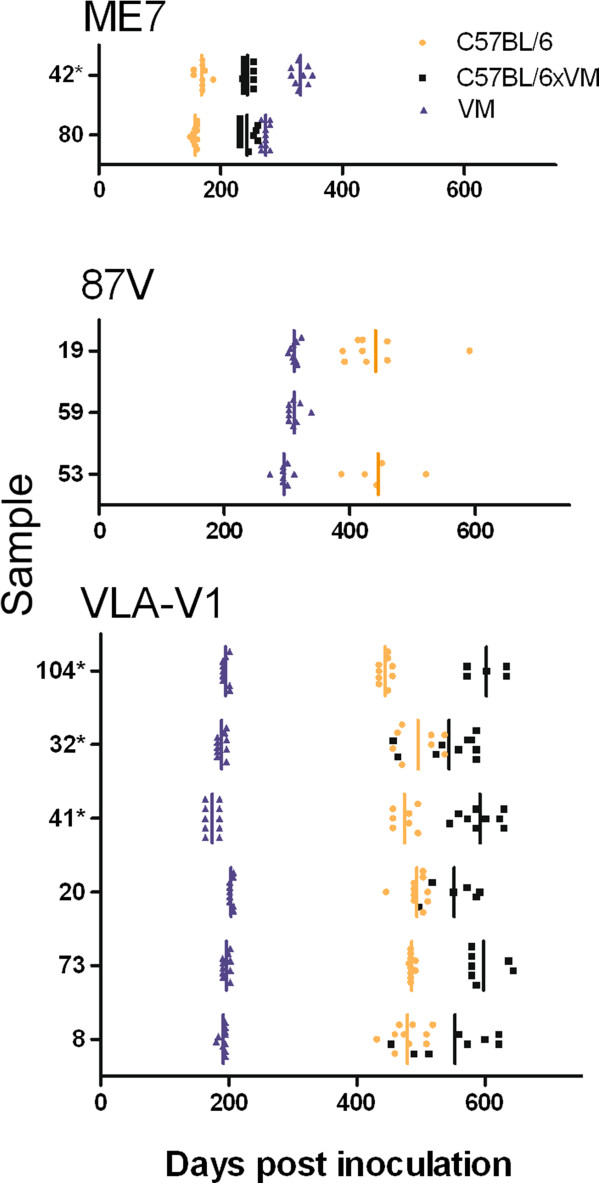
**Incubation periods (days post inoculation) of C57BL/6, VM and C57BL/6 × VM mice, following stabilisation of the agent through the VM line. **Samples with comparable IP have been grouped together. For each mouse line the IP for each individual mouse is represented along with the inoculum mean (vertical bar). (*) indicates that the sample originated in study 2.

**Figure 4:**
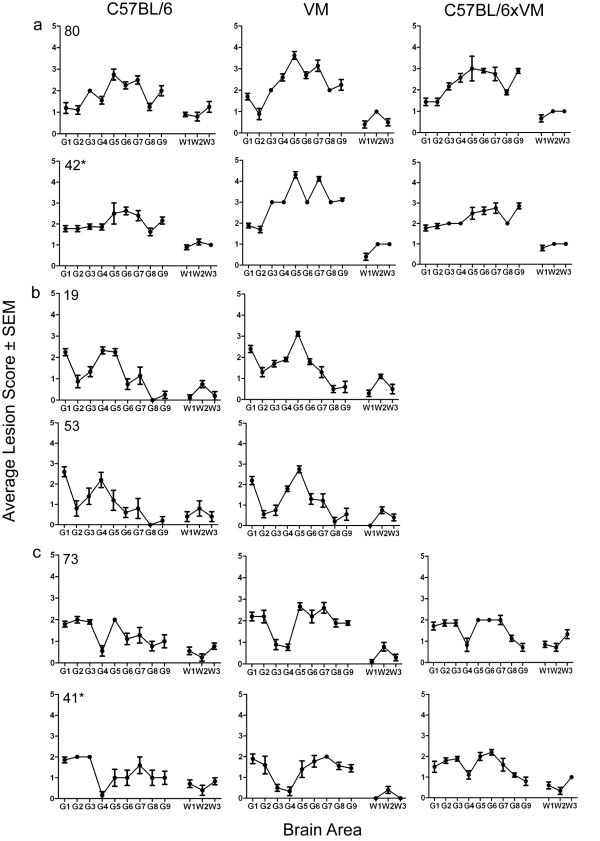
**Lesion profiles of C57BL/6, VM and C57BL/6 × VM mice, following stabilisation of the agent through the VM line. **In (**a**) examples of isolates displaying the ME7 LP are shown. In (**b**) and (**c**) examples of isolates displaying the 87V, and VLA-V1 LP, respectively, are shown. (*) indicates that the sample originated in study 2. Only clinically and histopathologically positive animals were included in the profile. Error bars indicate standard error of the mean.

Figure 3 shows the IP of 11 transmissions following stabilisation through the VM line and final characterisation in C57BL/6, VM and C57BL/6 × VM mouse lines. Five sources gave rise to strains ME7 or 87V [7,8]. However, six of the 11 sources formed a separate group as the IP in C57BL/6 and C57BL/6 × VM mice was approximately 200 days longer than in VM mice. This IP data bore similarities to the SCR11 strain in a previously published study, where LP were not available, owing to an insufficient number of clinically positive mice [9]. This was contrary to the present study where the number of TSE positive mice showing clinical signs permitted lesion profiling. However, the LP observed instead shared some similarities with strain SCR9 in VM mice [9] (Figure 4), although the IP in C57BL/6 and C57BL/6 × VM mice were not consistent with this strain. Since these sources did not collectively fit with the LP and IP of SCR9, SCR11 or any known strain isolated from natural classical scrapie cases, they were termed VLA-V1.

For sources 19 and 53 LP were only plotted for the C57BL/6 and VM lines where there were at least 5 clinically and histopathologically positive mice.

### Western blot analysis of C57BL/6 and VM mice

**Figure 5:**
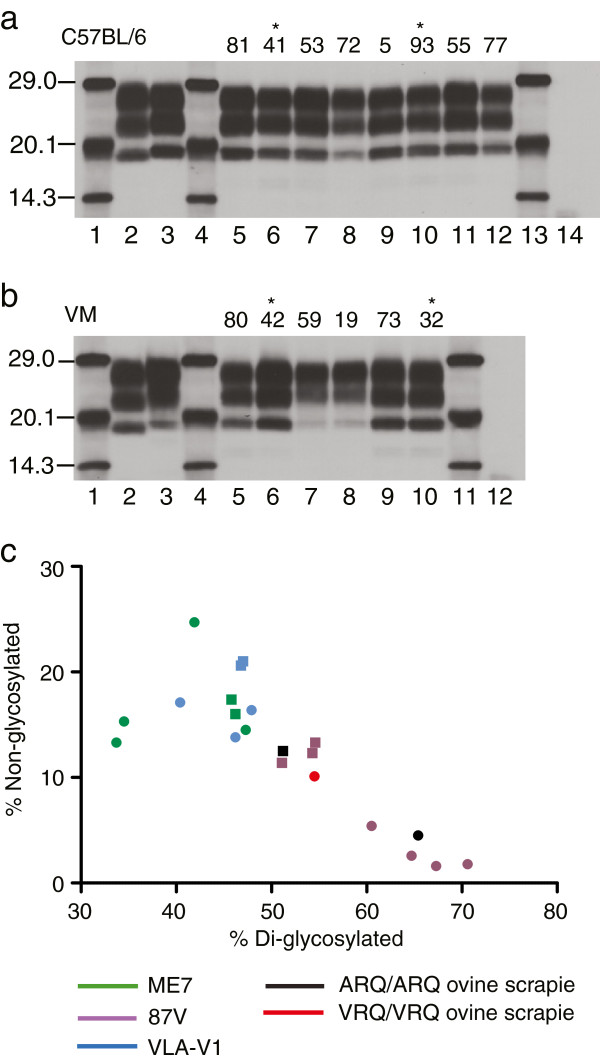
**Western blot analysis of proteinase K treated PrP**^**Sc**^**in C57BL/6 and VM mice detected with Sha31 antibody. **(**a**) Compares the molecular profile of different classical scrapie strains as defined by IP and LP analysis following stabilisation in C57BL/6 mice: Lane 2, ovine classical scrapie; Lane 3, ME7 in C57BL/6 control; Lanes 5–6, ME7 mice; Lanes 7–8, 87A mice; Lanes 9–10, 221C mice; Lanes 11–12, VLA-C1 mice; Lane 14, unchallenged C57BL/6 mouse; Lanes 1, 4 and 13, molecular mass markers. (**b**) Compares the molecular profile of different classical scrapie strains as defined by IP and LP analysis following stabilisation in VM mice: Lane 2, ovine classical scrapie; Lane 3, 87V in VM control; Lanes 5–6, ME7 mice; Lanes 7–8, 87V mice; Lanes 9–10, VLA-V1 mice; Lane 12, unchallenged VM mouse; Lanes 1, 4 and 11, molecular mass markers. (*) indicates that the sample originated in study 2. (**c**) Glycoform profiles of challenged VM mice: comparing relative band intensity of di- versus non-glycosylated PrP^Sc^. Results represent molecular profiles for individual animals taken from two experiments: squares, experiment 1; circles, experiment 2.

For each strain identified by IP/LP, two mice were selected from the C57BL/6 and VM mouse lines (representing the median IP), for Western blot analysis following proteinase-K digestion with Sha31 anti-PrP antibody (Figure 5). Results showed that in C57BL/6 mice there was no difference between the classical scrapie strains identified with regards to the electrophoretic mobility or glycoform ratio of the PrP^Sc^ bands (Figure 5a). All samples appeared to give the same molecular profile as the ME7 in C57BL/6 positive control sample. The 87A samples did have a reduced PrP^Sc^ concentration compared to ME7, 221C and VLA-C1 samples. A lower dilution of the 87A samples was required to achieve comparable band intensities.

In VM mice all strains gave the same molecular mass profiles. However, whilst ME7 and VLA-V1 gave the same glycoprofile and were indistinguishable, 87V samples differed and in turn, shared similarities with the 87V in VM positive control sample (Figure 5b). As with 87A the PrP^Sc^ concentration in these three samples was also reduced and lower dilution of samples was required to achieve comparable band intensities with other strains isolated in VM mice. These characteristics of 87V were confirmed in a further experiment where three additional VM mice were selected per scrapie strain for Western blot. 87V brains gave a significantly higher relative di-glycosylated band intensity and lower non-glycosylated band intensity respectively, (*p* < 0.05: One-Way ANOVA and post-hoc Tukey test) compared to VM mice challenged with other scrapie strains (Figure 5c).

### IHC analysis

**Figure 6:**
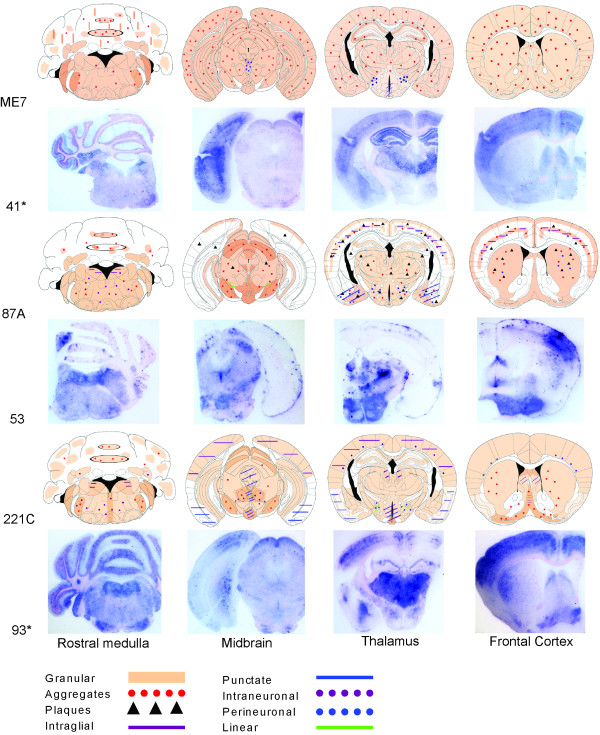
**Representative brain maps and PET-blots following stabilisation through the C57BL/6 line. **The predominant PrP^Sc ^types and deposition patterns associated with each identified strain are shown. For cerebellum maps, deposition that was observed throughout the granular layer is depicted within the ellipses outlined in black whilst the remaining space represents the molecular layer. (*) indicates that the sample originated in study 2.

**Figure 7:**
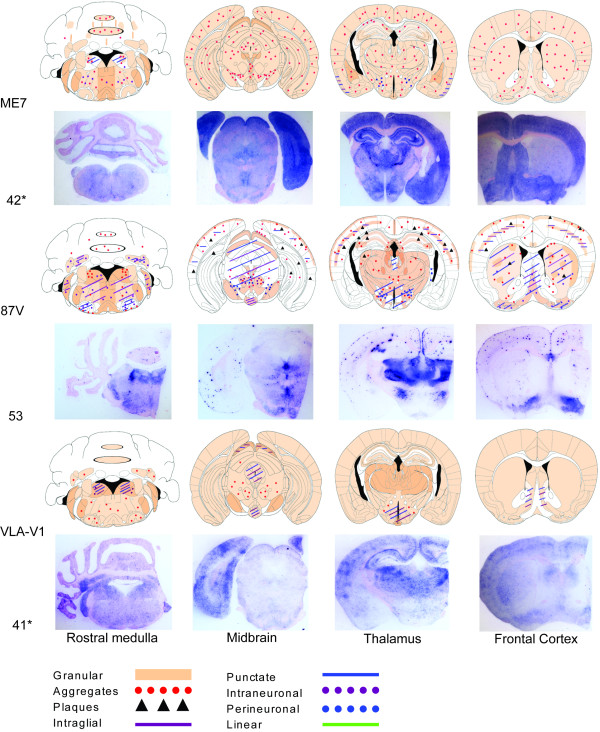
**Representative brain maps and PET-blots following stabilisation through the VM line. **The predominant PrP^Sc ^types and deposition patterns associated with each identified strain are shown. For cerebellum maps, deposition that was observed throughout the granular layer is depicted within the ellipses outlined in black whilst the remaining space represents the molecular layer. (*) indicates that the sample originated in study 2.

**Figure 8:**
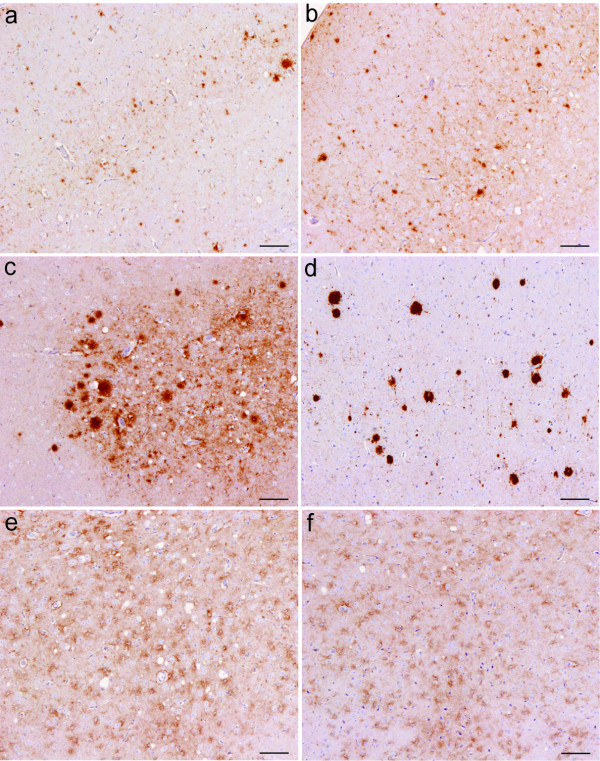
**Representative images of predominant PrP**^**Sc**^**deposition types associated with each strain in C57BL/6 and VM mouse lines. **(**a** and **b**) Aggregates of varying size associated with ME7 in C57BL/6 and VM lines, respectively; (**c**) fibrillar plaques associated with 87A in C57BL/6 mice; (**d**) large dense aggregates observed with 87V in VM mice; (**e** and **f**) “mottled” deposition in the thalamic nuclei associated with 221C and VLA-V1 in C57BL/6 and VM mice, respectively. Scale bars represent 100 μm.

The predominant PrP^Sc^ deposition patterns associated with each scrapie strain after stabilisation through either C57BL/6 or VM mouse lines are represented by brain maps in Figures 6 and 7. Of the two sources that were grouped as “VLA-C1” following IP and LP analysis, IHC revealed that source 55 gave a PrP^Sc^ deposition pattern that was consistent with ME7 in this mouse line whilst source 77 was comparable with 221C. The PrP^Sc^ deposition pattern in all remaining groups was consistent both within and between sources. Strain ME7 was identified in C57BL/6 (Figure 6) and VM (Figure 7) mice and the PrP^Sc^ deposition patterns found in both mouse lines were highly comparable. Throughout all coronal sections a widespread low level of granular deposition was observed within the neuropil together with many aggregates, varying in size but generally small and with an indistinct border (Figure 8a and 8b). Within the cerebellum the granular layer was affected, as could be the molecular layer but less extensively. The white matter and Purkinje cell layers remained relatively spared. Highly characteristic to this strain were fine, linear “streaks” of deposition observed in the molecular layer, generally perpendicular to the granular layer. In the hippocampus, ME7 predominantly targeted the polymorph layer of the dentate gyrus and stratum lucidum, the CA3 field and the pyramidal layer with granular labelling and small aggregates. However, other layers could also be minimally affected. In VM mice, the corpus callosum was more frequently targeted with aggregates. In both mouse lines perineuronal labelling was commonly found in the lateral hypothalamic nuclei.

Strain 87A was identified in C57BL/6 mice (Figure 6) whilst 87V was identified in VM mice (Figure 7). The PrP^Sc^ deposition patterns associated with each strain bore some degree of similarity in terms of location of PrP^Sc^ and in the types of deposition observed. Indeed of the strains isolated, both exhibit the greatest array of deposition types including plaques. In general, both show a background of granular staining within the neuropil throughout the medulla including the cochlear and cerebellar nuclei (however, notably, the facial nuclei were relatively spared with 87V), the midbrain (more extensively in 87A than 87V), all nuclei of the hypothalamus and thalamus and specific areas of the frontal level including the vertical limb of the diagonal band, ventral pallidum including medial forebrain bundle and islands of Calleja, the caudate putamen and lateral accumbens shell. Within the cerebellum the granular layer was most prominently affected with granular deposition (more so for 87A than 87V) and relatively large diffuse aggregates of PrP^Sc^ (87A), or smaller more defined aggregates (87V). Similar deposition was noted less extensively in the molecular layer. Intraneuronal labelling in the medulla was more prominent with 87V and extended to the cerebellar nuclei. Aggregates and plaques were a common and extensive feature of both 87A and 87V at all coronal levels. In 87A the plaques were of fibrillar type, exhibiting a dense core and radiating fibrils (Figure 8c). However, associated with 87V, there were large, round dense aggregates, some of which may represent primitive plaques in this mouse line as previously described [26,27] (Figure 8d). In the hippocampus there was consistent targeting with granular labelling of the molecular layer of the dentate gyrus and CA2 field with 87A. Conversely with 87V granular labelling was targeted predominantly along the fissure and CA2 field, whilst low level punctate labelling was observed particularly in the polymorph layer of the dentate gyrus and plaques/large aggregates were dispersed particularly within areas of granular staining, along the pyramidal layer and sometimes within the corpus callosum. The cortex was generally similarly affected by 87A and 87V: a double layer of granular labelling was evident affecting layers I (molecular layer) and V (internal pyramidal layer). Intraglial and punctate labelling was also found along layer V.

Strain 221C was identified in C57BL/6 mice (Figure 6). Predominantly widespread, granular PrP^Sc^ deposition was observed for this strain, with a degree of targeted intraneuronal, perineuronal, intraglial and punctate labelling across each coronal section, as shown. A few small aggregates were present mostly in the trigeminal nuclei, the granular and molecular layers of the cerebellum, ventral midbrain and within the vertical limb of the diagonal band, ventral pallidum and caudate putamen of the frontal level. Granular labelling was less intense in the lateral entorhinal cortex at the midbrain level and in the piriform cortex at the thalamic and frontal levels. The granular labelling, particularly in the thalamic nuclei and in the cortex could appear “mottled”, as darker shadows around glial cells (Figure 8e). In the hippocampus, granular labelling was predominantly targeted to the molecular layer of the dentate gyrus and the CA2 field whilst mildly targeted to the oriens layer and the stratum radiatum.

Strain VLA-V1 was identified in VM mice (Figure 7) where the PrP^Sc^ deposition pattern was very similar to that of 221C, although with even more widespread granular deposition. The mottled appearance of granular deposition, particularly within the thalamic nuclei and the cortex, was again evident (Figure 8f).

### PET-blot analysis

In this study PET-blots of the four coronal brain sections associated with each classical scrapie strain were presented in conjunction with the corresponding IHC brain maps for C57BL/6 (Figure 6) and VM (Figure 7) passaged mice. Although certain strain specific characteristics such as the plaques and large aggregates associated with 87A/87V or the ‘mottled’ appearance of the granular labelling in the thalamus of VLA-V1 mice were still evident on PET-blots, they lacked the high degree of architectural and cellular detail of the brain that could be observed using the IHC method. However, when compared with the corresponding brain maps, results show that the PET-blots clearly demonstrated the same general PrP^Sc^ distribution as observed using the IHC methodology and this was particularly well demonstrated by imaging of PET-blots on low power (Figures 6 and 7).

## Discussion

Classical scrapie appears to consist of several strains which have most likely evolved gradually under the influence of different ovine *PRNP* genotypes and other yet unknown factors [28]. It is important that distinct classical scrapie strains can be properly identified, both as a means of monitoring their presence in the national flock and in identifying emerging strains with possible zoonotic potential. As there is evidence of an association between ovine genotype and predilection for classical scrapie strains [28-30], it is also important to determine whether schemes that alter the genetic background of the flock change the repertoire of strains.

Ideally strain typing would be possible in the natural host however attempts to link phenotype with different strains have been met with limited success because it is difficult to isolate single entities of the agent from the original host. Whilst it is not currently possible to correlate with certainty different disease phenotypes with a given classical scrapie agent in the natural host, previous studies have suggested that TSE phenotype diversity in mice may reflect strain diversity in sheep [31]. However, other parameters such as PrP genotype may also play a significant role [28].

Applying the traditional methodology of IP and LP analysis, three strains were identified following bioassay of natural scrapie cases through each of the C57BL/6 and VM mouse lines including ME7, 87A and 221C through the C57BL/6 line [8,9] and ME7 and 87V through the VM line [7]. Notably the IP characteristics of 87V in two out of three isolates in the current study differed to a previous study [8] where clinical disease in C57BL/6 or C57BL/6 × VM lines was either longer than 700 days or exceeded the lifespan of the mice. A third previously unidentified strain, termed VLA-V1 was also observed in VM mice from over half of the scrapie sources with a prolonged IP of over 400 days in both C57BL/6 and C57BL/6 × VM mice. ME7 was observed in both mouse lines, where both the LP and the absolute and relative IP of each mouse line infected with this strain were remarkably consistent, irrespective of the mouse line through which the strain was stabilised. In accordance with the protein only hypothesis [1] strains are said to manifest in the tertiary conformation of the prion protein and the ability for a strain to replicate in a given host depends on the conformational flexibility of the host PrP^C^ molecule to adopt the conformation of a specific strain [32]. The ME7 strain may therefore represent a more stable conformation [17] and may not be influenced by host factors to the extent that other strains may be. Indeed ME7 is reportedly the most prevalent strain to have been isolated from natural scrapie sources both pre- and post- the BSE epidemic [9,33].

It has been previously reported that the deposition pattern of PrP^Sc^ across the brain of infected mice varies according to the agent strain [10-12], although the molecular basis of this is not yet fully known. Strain specific differences in PrP^Sc^ conformation may reasonably evoke differences in neuronal targeting, trafficking and processing/degradation of the protein [12,34-36]. In the current study IHC was used to characterise the identified classical scrapie strains according to the specific types and neuroanatomical location of PrP^Sc^. In order to demonstrate the predominant topographical distribution and PrP^Sc^ type associated with each strain a comparative whole brain mapping method was employed. This approach has been used previously to describe the PrP^Sc^ distribution in wild-type mice following primary passage of classical scrapie sources [10] and second passage of BSE [37], although in both studies only general neuropil deposition and aggregates and plaques were recorded. In a previous study the predominant PrP^Sc^ deposition types associated with ME7 and 87V were described [12]. Here we have expanded the description of each strain to account for 8 specific forms of PrP^Sc^ accumulation (granular, aggregates, plaques, intraglial, punctate, intraneuronal, perineuronal and linear) in conjunction with the whole brain mapping approach, thus increasing the discriminatory power of this method to distinguish different strains. A distinct advantage of this method is that it is less affected by titre and patterns can be identified on an individual mouse basis, in contrast with traditional IP and LP approaches which are based on mean data of all mice inoculated with a given source. Thus with the IHC method it is possible to compare the PrP^Sc^ pattern in mice associated with a given TSE strain both within and between inocula derived from different sources.

Each of the strains identified in the current study gave distinct PrP^Sc^ deposition patterns: ME7 through both C57BL/6 and VM mouse lines gave a very similar pattern of predominantly widespread granular deposition within the neuropil and the presence of many small aggregates. The characteristic perpendicular linear streaks observed were in agreement with those reported in previous IHC [12] and PET-blot [14] studies with this strain and are proposed to be associated with purkinje cells dendrites [12]. 87A through the C57BL/6 line and 87V through the VM line were the only strains to present with many large plaques and aggregates. 221C was associated with a widespread, diffuse granular deposition. The PrP^Sc^ pattern for VLA-V1 was very similar but had not been previously observed in association with any other strain in this mouse line.

In an earlier study the PrP^Sc^ patterns following transmission of classical field scrapie sources to wild-type mice were reported [10]. Notably patterns were detected on primary isolation that share great similarity with those reported in the current study. For example an ME7 PrP^Sc^-like pattern was reported in RIII and C57BL/6 mice, which suggests that this parameter stabilises before IP and LP. If used on primary passage IHC may therefore better reflect the repertoire of strains in the host and reduce the number of murine passages required for strain typing. IHC can also help to resolve strain identification when IP and LP are uninformative, even after full characterisation as was shown for VLA-C1 in the current study. Conversely this method may help to establish identification of novel strains, i.e. VLA-V1.

The PET-blot method has previously been proven to be a highly sensitive method for PrP^Sc^ detection. It has been reported that in C57BL/6 mice inoculated with ME7, PrP^Sc^ was detected in the brain by PET-blot 30 days after inoculation and 145 days before clinical signs [13], with greater sensitivity than both IHC and Western blotting. In sheep, PET-blot has been recently used to demonstrate the differential routes of spreading of PrP^Sc^ across the brain associated with different prion types [38] and as a method of discriminating classical scrapie and experimental BSE [24]. For strain typing, the PET-blot method was used previously in C57BL/6 mice to compare the distribution of BSE from different species with ovine and mouse adapted scrapie [14]. In the current study PET-blots proved a useful tool to clearly demonstrate the differences in neuroanatomical location of PrP^Sc^ associated with different classical scrapie strains and in this respect correlated well with IHC staining. In agreement with previous studies, IHC showed comparably better microscopic resolution at the cellular and subcellular levels, enabling different PrP^Sc^ types to be more clearly defined, although certain strain specific markers, i.e. perpendicular streaks of PrP^Sc^ through the molecular layer of the cerebellum, associated with ME7, were also identifiable by PET-blot as were denser structures including aggregates and plaques.

According to our data, Western blotting cannot be utilised to strain type classical scrapie following passage in wild-type mice: it was not possible to distinguish the scrapie strains identified in C57BL/6 mice by Western blotting whilst in VM mice only 87V could be distinguished from ME7 and VLA-V1. This was in agreement with a previous study [16] where 87V gave a highly glycosylated profile in VM mice compared to ME7. Indeed with the exception of 87V, the electrophoretic mobility and glycoform ratio of each strain identified was consistent, irrespective of the mouse line. However, Western blot may be a very useful phenotypic characteristic in helping to discriminate BSE from classical scrapie in wild-type mice or distinguishing TSE strains in transgenic mice, usually in conjunction with IHC [18].

In conclusion we have characterised 29 transmissions of classical scrapie field sources through the wild-type mouse bioassay and attained a comprehensive phenotypic description of each identified strain based on standard and alternative methodologies. Numerous scrapie strains have been reported using the wild-type mouse bioassay [5] however, only a very small sample of sheep scrapie cases have been thoroughly tested (20 pre-1985, 10 post-1985) [39]. The largest published study of natural classical scrapie isolates characterised through the mouse bioassay detailed transmissions of ten individual cases [9], although some of these previously reported cases originated from closed experimental flocks [40]. Additionally many of the mouse-passaged strains that have been described were derived from experimentally infected animals and may not reflect, either in terms of identity or relative prevalence, the classical scrapie strains which are endemic in the UK national flock [5,8].

The current study will serve as a standard reference which will be used to further analyse the dynamics of strain evolution and adaptation of classical scrapie through serial passage in wild-type mice, including the identification of strain specific parameters which can be used to identify strains during primary passage. Upon identification of the strains from at least 100 field cases of classical scrapie using the methodology presented here, we will attempt to analyse the strain demographics of classical scrapie in the UK and identify host parameters that may influence them. These data suggest that analysis of these sources should include whole brain mapping based on the distribution of different PrP^Sc^ types as revealed by IHC on individual mice which may negate the requirement for serial passages currently required for strain typing in mice and in addition, may reveal more than one phenotype emerging from a single source.

## Competing interests

The authors declare that they have no competing interests.

## Authors’ contributions

KB participated in lesion profiling, analysed IHC data and drafted the manuscript. CMV participated in lesion profiling and IHC analysis. RL participated in experimental design. TH, LT and LAT performed and analysed Western blotting data. MD and PW performed IHC and PET-blots. MMS conceived and designed experiments. JS conceived and designed experiments, participated in all data analysis and drafted the manuscript. All authors reviewed and approved the final manuscript.
